# Development and validation of a deep learning model for improving detection of nonmelanoma skin cancers treated with Mohs micrographic surgery

**DOI:** 10.1016/j.jdin.2023.10.007

**Published:** 2023-11-07

**Authors:** Eugene Tan, Sophie Lim, Duncan Lamont, Richard Epstein, David Lim, Frank P.Y. Lin

**Affiliations:** a Western Skin Institute, Melbourne, Australia; b Skintel, Auckland, New Zealand; c Alfred Health, Melbourne, Australia; d Department of Pathology, Waikato Hospital, Hamilton, New Zealand; e School of Medicine, University of New South Wales, Sydney, Australia; f Kinghorn Centre for Clinical Genomics, Garvan Institute of Medical Research, Sydney, Australia; g NHMRC Clinical Trials Centre, University of Sydney, Camperdown, NSW, Australia

**Keywords:** artificial intelligence, basal cell carcinoma, deep learning, digital pathology, Mohs micrographic surgery, squamous cell carcinoma

## Abstract

**Background:**

Real-time review of frozen sections underpins the quality of Mohs surgery. There is an unmet need for low-cost techniques that can improve Mohs surgery by reliably corroborating cancerous regions of interest and surgical margin proximity.

**Objective:**

To test that deep learning models can identify nonmelanoma skin cancer regions in Mohs frozen section specimens.

**Methods:**

Deep learning models were developed on archival images of focused microscopic views (FMVs) containing regions of annotated, invasive nonmelanoma skin cancer between 2015 and 2018, then validated on prospectively collected images in a temporal cohort (2019-2021).

**Results:**

The tile-based classification models were derived using 1423 focused microscopic view images from 154 patients and tested on 374 images from 66 patients. The best models detected basal cell carcinomas with a median average precision of 0.966 and median area under the receiver operating curve of 0.889 at 100x magnification (0.943 and 0.922 at 40x magnification). For invasive squamous cell carcinomas, high median average precision of 0.904 was achieved at 100x magnification.

**Limitations:**

Single institution study with limited cases of squamous cell carcinoma and rare nonmelanoma skin cancer.

**Conclusion:**

Deep learning appears highly accurate for detecting skin cancers in Mohs frozen sections, supporting its potential for enhancing surgical margin control and increasing operational efficiency.

## Graphical abstract

**Figure:**
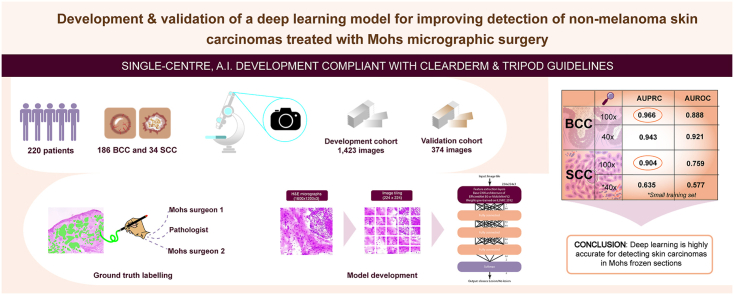

**Capsule Summary**

• Mohs surgery is an effective procedure for nonmelanoma skin cancers but remains labor-intensive, time consuming, and costly.
• Deep learning facilitated margin-control appears to be a valuable addition to Mohs surgery, given its potential for improving resectional efficacy and reliability while minimizing extent, duration, and cost.

## Introduction

Mohs micrographic surgery (MMS) is the recommended procedure for local high risk basal cell carcinomas (BCCs) and squamous cell carcinomas (SCCs).^1^^,^^2^ Although excellent concordance exists between MMS surgeons and dermatopathology,^3^^,^^4^ interobserver discordance may still occur.^5^ Variability is seen even amongst experienced surgeons particularly in complex tumors with challenging pathology.^6^ As Mohs surgeons often operate in an individual or a small group setting,^7^ variable interpretation of frozen sections may also arise due to operator fatigue and/or inconsistent techniques of tissue preparation.

Deep learning has permeated various fields of medicine^8^, ^9^, ^10^ and has demonstrated exceptional performance in the diagnosis of skin diseases on clinical and dermoscopic images.^11^, ^12^, ^13^, ^14^, ^15^, ^16^ It also has the potential to assist Mohs surgeons in optimizing intraoperative margin control through reduction of interobserver discordance^17^ but few studies have examined the application of deep learning on Mohs frozen section images.^18^

Here we report on the development of deep learning models that detect nonmelanoma skin cancers (NMSCs) on frozen sections obtained during MMS. These models were trained on a dataset consisting of archival microscope images and routine diagnostic setups and procedures. The primary objective was to validate these models develop models as a first step towards the longer-term objective of enhancing Mohs surgical workflow.

## Methods

### Study design

This study for diagnostic model development was approved by the St. Vincent's Hospital Human Research Ethics Committee (2021/ETH00647). This research is reported in accord with CLEAR Derm Consensus guidelines for artificial intelligence (AI) algorithm reports in dermatology^19^ and Transparent Reporting of a multivariable prediction model for Individual Prognosis Or Diagnosis statement^20^ (Supplementary Tables I and II, available via Mendeley at https://data.mendeley.com/datasets/fh7sk5ksmk/2).

### Study population

All patients who had undergone MMS for NMSC in 2015 to 2021 at a Mohs surgical unit in Melbourne, Australia and consented to research during surgery, were identified. The cases were included with diagnostic labels assigned based on the tumor observed during Mohs surgery.

#### Cohort and image data

Archival images of skin lesions from patients who underwent surgery prior to the study's conception (February 20, 2015-December 1, 2018) were used for model development (development cohort). Patients who had been seen after the index date (January 24, 2019-December 6, 2021) were used for testing model performance (validation cohort). Nonidentifiable demographic data were collected for the cohort characterization.

All pathology slides were prepared using a protocol of progressive Mayer’s hematoxylin and eosin stain^21^ on Epredia Linistat Linear Stainer, Fisher Scientific Pittsburgh, PA 15,275. Images of focused microscope view (FMV) were acquired using Leica ICC50 W, 5.0-megapixel Camera attached to DM1000 microscope and captured via the Leica software (LAS-EZ v3.0). Full color images (at 1600 × 1200 pixels resolution) were acquired at 40× and 100× magnification levels. The diagnosis was made by the lead author at the time of surgery. Air bubbles and freezing artifacts were unprocessed during model training to enhance procedural generalizability.

#### Image processing and training of deep learning models

The pipeline was designed to localize regions of malignant lesions on digitized images of FMV by producing an overlaying saliency map indicating the probability of containing tumor. Detection of tumor was performed through a tile-based classification method that utilized the sliding prediction window technique. By incorporating overlapping predictions, the sliding window technique aims to produce more accurate contours for the predicted regions and minimize any potential omissions. All models were based on a convolutional neural network architecture, utilizing the feature extraction layers from EfficientNet B0^22^ or MobileNetV2^23^ models concatenated with 3 dense layers followed by a final softmax layer, employing a categorical cross-entropy loss function (Supplementary Fig 3, available via Mendeley at https://data.mendeley.com/datasets/fh7sk5ksmk/2). The feature extractors pretrained on ImageNet were obtained from Tensorflow Hub for weight initialization before full fine-tuning. Image augmentation, early stopping, and limiting to 200 epochs were used during training to prevent overfitting; separate models were trained at 40× and 100×.

#### Primary and exploratory analyses

Model set 1 - In this primary analysis, fully supervised learning models were trained on the images with tumor locations manually segmented by an experienced Mohs surgeon (E.T.). All models were trained on split tiles of 224 × 224 pixels. Each square tile was labeled as “positive” if the tumors occupied ≥10% of the area or categorized as controls. Extra controls were included from images of other diagnoses (eg, SCC to train BCC models and vice versa, actinic keratoses and normal skin).

To ensure that ground truth was reproducible, the concordance was estimated by comparing the segmentation masks prepared by 2 experienced Mohs surgeons (E.T.; D. Lim) and an anatomical pathologist (D. Lamont) on randomly selected 25 images. Interrater agreement was measured by Fleiss' kappa for identifying ≥5% tumor presence in each 50 × 50 pixel tile generated from the masks.

Model set 2 - Considering the labor-intensive manual segmentation process, we further evaluated in an exploratory analysis on whether models trained through weakly supervised learning (WSL) can achieve similar performance in region of interest detection in the subset of BCC 10× images; WSL has been known to achieve clinical-grade precision without requiring human annotations.^24^ In this 2-stage approach, classifiers were first trained using all tiles labeled with the top-level diagnosis without expert annotations. The second stage of the training selectively included only the tiles with high inferred probabilities from the first-step. The control image tiles remained unchanged during this process.

#### Performance metrics and validation studies

The primary performance metric was the pixel-level area under the precision-recall curve (AUPRC), estimating the average precision across all prediction thresholds for each tumor type at the 40× and 100× magnifications. Secondary metrics include the area under the receiver-operating characteristic curve (AUROC), the highest Dice coefficient, and folds enrichment of precision (AUPRC divided by the proportion of positive pixels). To accurately assess these metrics on the probability maps, the ground truth masks were reshaped to fill the same boundaries prescribed by the deep learning algorithm. Additional explanation is provided in Supplementary Fig 4, available via Mendeley at https://data.mendeley.com/datasets/fh7sk5ksmk/2.

The internal model validity was assessed on model set 1 using leave-one-out cross-validation (LOOCV) on the subset of images where the ground truth has been annotated. To avoid contamination of information from images obtained from the same patient, each test fold was limited to a single patient (patient-level LOOCV). The external validity was estimated on the temporal validation cohort and stratified by magnification levels and model architectures. The CIs were estimated using ordinary bootstrap resampling over 10,000 iterations. The paired Wilcoxon test was used to compare the metrics between models on the same images; the type I error rate was set at 0.01 adjusted for multiple testing.

#### Software

All models were built on the TensorFlow framework 2.7.0. The preprocessing and evaluation tools were implemented using customized computer scripts. Descriptive statistics and the bootstrapping procedures were performed using the R Statistical Environment 4.0.

## Results

### Cohort and tumor characteristics

Two hundred fifty-eight patients over 293 visits were screened. Digital FMV images for 220 patients were available and 1836 images were retrieved. The median age of the patient population was 61 years old (interquartile range, 58-76). One hundred eighty-seven patients (85%) were diagnosed with BCC, with nodular (*n* = 88), infiltrative (*n* = 49), and superficial (*n* = 20) being the commonest subtypes. Thirty-three patients (15%) with SCC were identified including 1 patient with perineural invasion (Table I). Three patients (1%) had metachronous BCC and SCC.

**Table I:** Characteristics of the development and validation cohorts (*N* = 220)

Clinical values	Development cohort (*n* = 154)	Validation cohort (*n* = 66)
Age group, y (%)		
<40	7 (5)	4 (6)
41-60	44 (29)	18 (27)
61-80	78 (51)	38 (57)
> 81	25 (16)	6 (9)
Gender		
Male	84 (55)	43 (65)
Female	70 (45)	23 (35)
Tumor type		
Basal cell carcinoma (BCC)		
Nodular BCC	58 (38)	30 (45)
Infiltrative BCC	32 (21)	17 (26)
Superficial BCC	17 (11)	3 (5)
Micronodular BCC	12 (8)	4 (6)
Morpheaform BCC	5 (3)	0 (0)
Basosquamous BCC	2 (1)	2 (3)
Nodulocystic BCC	2 (1)	1 (2)
Desmoplastic BCC	0 (0)	1 (2)
Squamous cell carcinoma (SCC)	26 (17)	8 (12)
Location of the tumor		
Nose	61 (40)	32 (48)
Ear	19 (12)	9 (14)
Cheek, forehead, and temple	44 (28)	11 (18)
Lip	11 (7)	2 (3)
Periocular, eyelid, and eyebrow	12 (8)	9 (15)
Scalp (frontal, vertex, or occipital)	5 (3)	0 (0)
Other	2 (1)	3 (6)
No. of Mohs procedures		
1	133 (86)	60 (91)
2	16 (10)	6 (9)
≥3	5 (3)	0 (0)
No. of images per patient, median (range)	7 (0-35)	6 (0-52)
No. (%) with annotated images	79 (51)	66 (100)
No. (%) of Mohs stages		
1	44 (29)	43 (65)
2	85 (55)	18 (27)
3	16 (10)	2 (3)
≥4	9 (6)	3 (5)

Most tumors were located on the nasal region (*n* = 93, 42%), with nasal ala and tips being the most frequent sites involved (*n* = 59, 27%) followed by the dorsum and sidewall (*n* = 27, 12%). A median of 1 surgical procedure per patient was performed (range 1-5), with the median number of MMS stages of 2 (range 1-8). The median size of lesions before MMS was 1.4 cm (range 0.2-4.5). The full cohort and tumor characteristics are shown in Table I.

**Fig 1:**
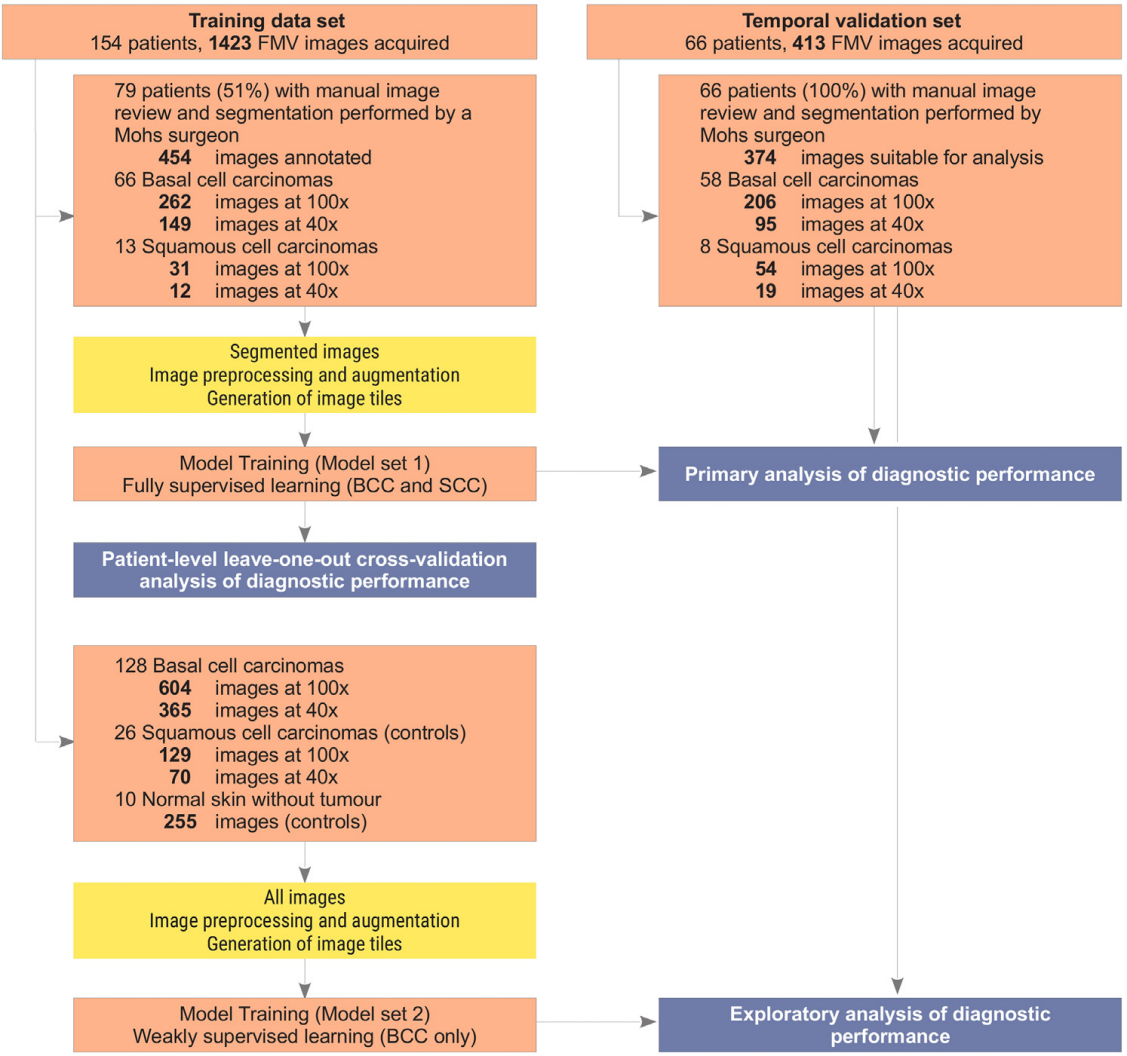
Flowchart of data analysis. *BCC*, Basal cell carcinoma; *FMV*, focused microscope view; *SCC*, squamous cell carcinoma.

Based on the date of the project initiation, 154 patients (70%) were assigned into the development cohort and 66 patients (30%) assigned to the temporal validation cohort (Fig 1). The validation cohort was similar to the training cohorts with respect to demographic, pathology, and tumor subtypes, except for the lower number of MMS stages per operation (*P* < .001, χ^2^ test).

### Concordance of tumor localization in frozen section images

Estimated using 25 FMV images (23 BCC and 2 SCC), the interannotator agreement between segmentation masks was high (mean Fleiss' Kappa 0.877, 95% CI 0.842-0.911). Each segmentation task required 20 to 30 minutes to complete. Further discussion on the concordance among specialists is presented in Supplementary Fig 5, available via Mendeley at https://data.mendeley.com/datasets/fh7sk5ksmk/2.

### Model development and cross-validation analysis

*BCC*—a total of 267 images at 100× magnification level in 66 (of 154) patients had been annotated, with 241 images (90%) in 59 patients containing ≥10% of the nontumor region that is considered suitable for LOOCV (59-fold) analysis. The median AUPRC were 0.939 (95% CI: 0.923-0.953) for EfficientNet and 0.949 (0.932-0.960) for MobileNet, equivalent to a median of 2.56 (2.45-2.71) and 2.67-fold (2.52-2.84) more likely to correctly locate the lesions than at random. Both architectures showed strong discrimination, as evidenced by high median AUROCs of 0.900 (0.873-0.923) and 0.917 (0.904-0.931, Supplementary Table VI, available via Mendeley at https://data.mendeley.com/datasets/fh7sk5ksmk/2); the corresponding median Dice coefficients were 0.892 (0.883-0.903) and 0.897 (0.888-0.907).

Highly concordant performances were seen at the 40× magnification level. In 147 of 149 images (99%) suitable for the LOOCV analysis, we observed high median AUPRCs of 0.944 for both EfficientNet and MobileNet, equivalent to a median of 4-fold enrichment of precision over the baseline. The median AUROC were 0.954 (0.942-0.970) and 0.948 (0.926-0.968), with median Dice coefficients of 0.899 (0.884-0.911) and 0.897 (0.870-0.909), for both architectures respectively (Supplementary Table VI, available via Mendeley at https://data.mendeley.com/datasets/fh7sk5ksmk/2).

*SCC*—in 23 patients with SCC, the segmented masks for 13 cases (31 images for 100x and 12 for 40x) were available for LOOCV. For 25 images (80%) containing ≥10% of the nontumor region, the AUPRC for the 100x models were 0.910 (0.878-0.963) for EfficientNet and 0.920 (0.888-0.980) for MobileNet. The median folds enrichment of precision were 2.66 (2.41-3.12) and 2.74 (2.28-3.30) respectively. The median AUROCs were 0.903 (0.795-0.939) and 0.912 (0.834-0.960), and the Dice coefficients were 0.929 (0.887-0.973) and 0.959 (0.878-0.973), for the 2 models (Supplementary Table VI, available via Mendeley at https://data.mendeley.com/datasets/fh7sk5ksmk/2).

Only 7 of 12 images were suitable for LOOCV at 40x. Moderate AUPRCs were achieved (median 0.795 for EfficientNet, 0.560-0.927, and 0.701 for MobileNet, 0.336-0.979). The respective AUROCs were 0.747 (0.678-0.877) and 0.768 (0.448-0.981).

### Performance of region of interest detection by model set 1 in temporal validation cohort

**Fig 2:**
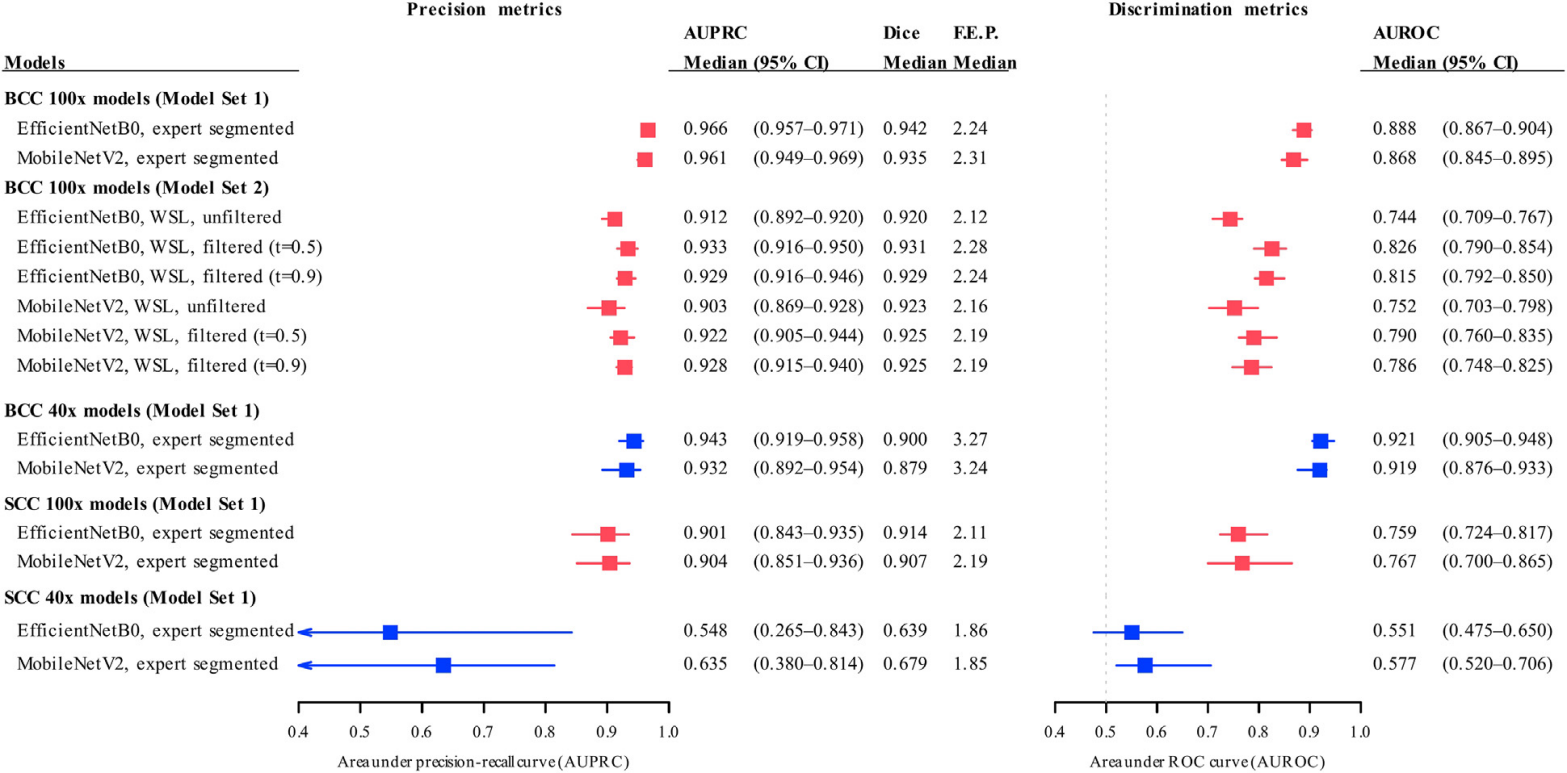
Performance of deep learning models stratified by model sets, architecture, training data, and training method. *AUPRC*, Area under the precision-recall curve; *AUROC*, area under the receiver operating characteristic curve; *BCC*, basal cell carcinoma; *Dice*, Dice coefficient; *FEP*, folds enrichment of precision; *SCC*, squamous cell carcinoma; *WSL*, weakly supervised learning.

**Fig 3:**
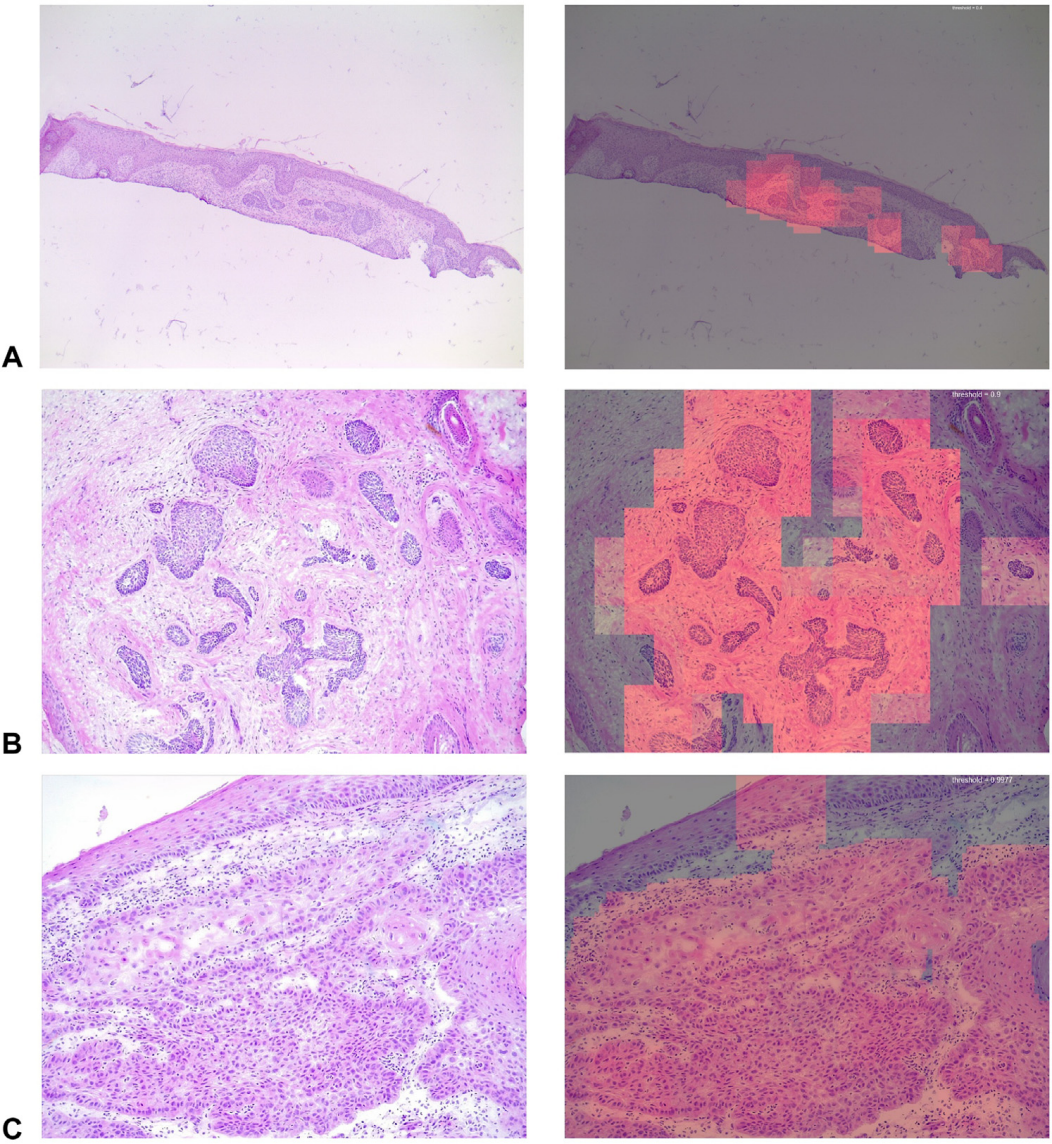
Example of probability map produced by the deep learning models for detection of nonmelanoma skin cancer in Mohs frozen sections. **A,** Nodular BCC, 40×, with saliency maps produced by EfficientNet B0 100× model (pixel level AUPRC: 0.826, AUROC 0.981). **B,** Micronodular BCC, 100×, MobileNet V2 100× model (AUPRC 0.996, AUROC 0.991). **C,** Squamous cell carcinoma, 100× model, MobileNet V2 (AUPRC 0.991, AUROC 0.912). All deep learning models were trained with full supervised learning (model set 1).

The validation cohort included 66 patients with NMSC with expert-annotated FMV images. In 58 patients with BCC (206 images for 100× magnification level; 95 for 40×), the saliency maps located BCC with high median AUPRC (0.966 for 100×, 0.957-0.972; 0.943 for 40×, 0.919-0.958) and AUROC (0.889 for 100×, 0.869-0.904 and 0.922 for 40×, 0.905-0.948). Per-image analysis revealed no performance difference between the EfficientNet and MobileNet at 100× (median difference in AUPRC 0.00013, *P* = .23; in AUROC 0.0021, *P* = .11, paired Wilcoxon's test), although higher precision and discrimination were seen in EfficientNet models at 40× (median difference in AUPRC 0.0069, *P* = .009; in AUROC: 0.016, *P* = .0034, Table II and Fig 2). Examples of the saliency maps are shown in Fig 3.

**Table II:** Performance of the best performing deep learning models for detecting regions-of-interest in the temporal validation cohort

Model and testing images	AUPRC	AUROC	Dice coefficient	FEP
Basal cell carcinoma (BCC, *n* = 58)				
100× images (206)				
100× model, median (CI)	0.966 (0.957-0.972)	0.889 (0.869-0.904)	0.942 (0.937-0.945)	2.31 (2.23-2.42)
40× images (95)				
40× model, median (CI)	0.943 (0.919-0.958)	0.922 (0.905-0.948)	0.900 (0.875-0.918)	3.27 (2.7-4.06)
100× model, median (CI)	0.843 (0.801-0.897)	0.897 (0.871-0.915)	0.794 (0.763-0.839)	3.35 (2.84-4.33)
Squamous cell carcinoma (SCC, *n* = 8)				
100× images (54)				
100× model, median (CI)	0.904 (0.851-0.936)	0.767 (0.700-0.865)	0.915 (0.849-0.93)	2.19 (2.12-2.49)
40× images (19)				
40× model, median (CI)	0.635 (0.38-0.814)	0.577 (0.520-0.706)	0.680 (0.532-0.918)	1.86 (1.3-2.02)

*AUPRC*, Area under the precision-recall curve; *AUROC*, area under the receiver operating characteristic curve; *FEP*, folds enrichment of precision.

High precision was also evident in the SCC model at 100× magnification (median AUPRC 0.904, 0.851-0.936) with moderate discriminability (median AUROC 0.767, 0.700-0.865). No differences were observed in AUPRC between MobileNet and EfficientNet (median difference in AUPRC −0.009, *P* = .10, and in AUROC −0.031, *P* = .03). Only modest AUPRC (median 0.536, 0.38-0.81) and AUROC (median 0.577, 0.520-0.706) was achieved by the best 40× models.

Resizing 40× BCC images to 100× resulted in similar level of discriminability (median AUROC: 0.897, 0.869-0.904, median pairwise difference −0.014 vs the 40× model, *P* = .21) but lowered precision (median AUPRC 0.843, 0.801-0.897, median difference −0.039, *P* < .001). The model performance was uniform across the BCC subtypes with >10 samples (Supplementary Table VII, available via Mendeley at https://data.mendeley.com/datasets/fh7sk5ksmk/2).

### Exploratory analysis on the temporal validation set for model set 2

The WSL models showed high precision in region of interest detection with an AUPRC >0.9, albeit with lower AUPRC and AUROC compared to models in model set 1 (Fig 3). For example, WSL models at the filtering threshold t = 0.5 showed lower discriminability in both EfficientNet (AUROC 0.826, median pairwise difference −0.042, *P* < .001) and MobileNet (AUROC 0.790, median difference −0.046, *P* < .001) architectures.

## Discussion

The central finding of this study is that deep learning locates NMSCs on digitized images of Mohs frozen sections with high accuracy and efficiency supporting the use of AI-assisted margin control as a “second read” to reduce human error.^5^

A recent study has shown a comparable performance from the whole slide images of BCC acquired using a digital slide scanner.^18^ Despite the commercial availability, the cost of scanners remains prohibitive to many clinics and not justifiable for low-volume centers. A software-assisted review of FMV images could be implemented in any Mohs laboratory at a fraction of the latter cost, reusing only inexpensive microscope systems. Such an AI approach would be expected to have maximum utility when integrated with a built-in slide scanner, in-effect eliminating the need to examine frozen section slides altogether though further evidence will be needed to corroborate the value and safety of this approach. Recent proof-of-concept studies support the findings of this study but are limited by the lack of evaluation of performance metrics in clinical cohorts.^24^, ^25^, ^26^

The present study provides several valuable insights into AI data quality. First, reviewing discrepancies among specialists is necessary to understand how the causes of over- or under-diagnosis occur during single-pass pathology review. For example, distinguishing between a hair follicle and tumor in BCC, or between inflammatory cells and tumor in SCC, can be challenging. In the real-world setting, the Mohs surgeon and pathologist may review multiple wafers or different pathologic levels. Transparent annotations are imperative in understanding the constraints of applicability of AI-based models, given that variations in diagnostic labels are likely to hinder the reproducibility of results.^27^

Second, although validations at external sites are planned, the work presented here supports the feasibility of site-specific diagnostic models based on reutilization of archival images from medical records. Third, our research highlights the importance of involving surgeons and pathologists in the creation of accurate ground truth annotations to achieve good classification results. This manual semantic segmentation is a tedious task that often acts as the rate-limiting step in model development due to its high-level of precision and the need for expert knowledge in making a diagnosis. Further investigations into alternative methods for automation, such as WSL, may overcome this limitation.

Our study has several limitations. Microscopic images were taken in a single Mohs unit; variations in surgical techniques, sample preparation, and imaging acquisition methods could thus impact model accuracy at other sites. Furthermore, only microscopic views at the standard resolution used by Mohs surgeons were analyzed; visualizing the complete section at high resolutions through whole slide analysis could improve margin control. Moreover, in contrast to the good performance of the BCC models, insufficient data hampered SCC model performance at 40x magnification in our study, highlighting the importance of adequate training data. Image sharing across Mohs units may facilitate collaborative model development for tumor types less frequently seen.

Immunohistochemical staining was not performed in this Mohs unit. Future studies should include this assessment, particularly given that there is increasing interest in managing melanoma with MMS. Lastly, our tile-based classification paired with custom evaluation metrics was designed to maximally leverage the 3-dimensional spatial context of tumors to identify hidden or unseen tumor areas. This spatial understanding is vital for Mohs surgery as unexpected inflammation or fibrosis may be a harbinger of tumor necessitating another stage.^28^^,^^29^ Future studies will benchmark our models against segmentation approaches like U-net,^30^ to also assess its effectiveness in prospective trials in overall margin control beyond accurate localization.

In conclusion, this study supports the accuracy of deep learning models for detecting NMSCs on Mohs frozen sections. Prospective studies in accordance with CONSORT-AI and SPIRIT-AI guidelines will determine the acceptable bar for adoption as a diagnostic support tool during real-time tumor removal.

## Data availability statement

The software code is made publicly available on GitHub repository (https://github.com/fpylin/MohsAI). The dataset can be obtained from the authors upon request, subject to additional reviews by relevant Human Research Ethics Committees and Institutional Review Boards.

## Conflicts of interest

None disclosed.
